# The Homœopathic Hospital, Great Ormond Street

**Published:** 1894-06-30

**Authors:** 


### HOSPITAL CONSTRUCTION.

### THE HOMŒOPATHIC HOSPITAL, GREAT ORMOND STREET.

This hospital is in course of reconstruction on the site of the former building, with the addition of the ground occupied by three adjacent houses. The work of the old hospital was carried on in several houses which were thrown together and altered from time to time to suit the growing needs of the work. This, at best but a makeshift arrangement, was found to be quite inadequate to the increasing demands upon it and extension was practically impossible. The committee therefore determined to start afresh, and upon a larger site to build a complete hospital upon modern lines.

The new building consists of one straight block fronting the street with three wings projecting out from the back. A portion of the old hospital has been left standing, and this, with the Nurses' Institute, erected in 1882, has been fitted up for the accommodation of 40 patients during the rebuilding. In the basement of this building the work of the out-patient department will also be carried on temporarily.

Commencing with the lowest floor of the new building, we find the basement occupied by the out-patient department. The patients enter by a staircase from the street-level, and, after passing the porter's office, find themselves in a large central waiting-hall, from whence they are drafted off either to the right or left, according to the nature of their cases, to the benches which occupy the wings of the waiting-hall. Here, on each side are two large consulting-rooms, two of which are provided with an examination-room and a dressing-room each, and two with an examination-room and a dressing-room in common. The latter rooms would appear from the plan to be unprovided even with a borrowed light. At one end of the waiting-hall is the dispensary, close to which is the exit for patients. In the centre of the waiting-hall, at the back, is a small refreshment bar. The w.c.'s for patients are placed in two wings at the back, and are approached through the open court-yard. The only communication with the upper part of the building is by a staircase at the side of the boiler-room opening into a lobby at the back of the waiting-hall. Thus the medical officers must cross the waiting-hall on their way to and from the consulting-rooms; an inconvenient arrangement, but it is not easy to see how it could have been avoided. It would have been better to have omitted the side walls of the lobby and put a roof only; the severance between the in and out-patient department would then have been perfect. A detached building in the rear contains rooms for disinfecting and for dirty linen. On the ground floor is the main entrance to the hospital. To the right of the entrance is the porter's office, and to the left is the casualty-room; at the back is the main staircase, in the well of which is a hydraulic lift for patients. These are all in a central block to east and west of which are wings of unequal lengths.

Adjoining the casualty-room is a bath-room. The west wing is occupied by the board-room, secretary's office, and clerks' office. The east wing contains a ward for fourteen beds, with a duty-room, bath-room, and sanitary offices. Want of space has necessitated an arrangement of a somewhat unusual kind in the bath-rooms. When the bath is in use, the partition which forms one side of the passage to the w. c.'s has to be folded back in order to give access to the bath. When, therefore, the bath-room is occupied, access to the w. c.'s is cut off.

The sanitary towers, which are approached by covered bridges, are provided each with two w.c.'s fitted with Hellyer's "Bracket" apparatus, a stop sink (the McHardy type), and two lavatory basins. The floors of the wards are of solid oak blocks; the ward grates are Boyd's Hygiastic ventilating stoves with descending flues carried to a vertical shaft on the north wall. At the back, over the boiler-room, is the medical officer's room. This latter communicates by an open iron bridge with the post-mortem room and the pathological room, which are over the disinfecting room, &c. Adjoining the post-mortem room is a mortuary chamber, which will at some future time be connected by an open bridge with a waiting-room for friends. Bodies will be taken to the mortuary on a rolling-way from the hall close to the lift.

The first floor contains two wards, one for eight beds, the other for fourteen beds. In the central block is a sitting-room and bed-room, and at the back is the operation-room. This room is provided with three large north windows, but no top-light.

The second floor is similar to the last, but over the operation-room is the nurses' dining-room, with a combined bath-room and w.c. adjoining.

The third floor is also similar in all respects, expect that the space over the nurses' dining-room and bath-room is occupied by the resident medical officer's room, with a combined bath-room and w.c. adjoining.

The obvious difficulties arising from the cramped nature of the site to some extent disarm criticism, but it is to be hoped that some way will be found to alter this very objectionable arrangement of combining a w.c. and a bath in one room.

The fourth floor contains in the east wing the kitchen offices, servants' hall, and stores. In the central part is a bed-room and sitting-room and a store-room. In the western wing are special rooms for private surgical operations, with a nurses' room attached. At the back is an isolation ward and the linen-room. The central portion and back wing are carried up a storey higher, and contain bed-rooms and a bath-room for servants.

The planning of the building is, as a whole, most ingenious, and reflects great credit on Mr. William A. Pite, the architect. It is evident that Mr. Pite has not been above taking lessons from what has been done in recent hospital work, and has applied the knowledge thus gained with ability and discretion. He has had a difficult task, and has accomplished it with success. We shall look forward with much interest to seeing the completed building.

